# Effects of a rainwater harvesting system on the soil water, heat and growth of apricot in rain-fed orchards on the Loess Plateau

**DOI:** 10.1038/s41598-024-58667-7

**Published:** 2024-04-23

**Authors:** Na Feng, Yan Huang, Jiao Tian, Yongliang Wang, Yi Ma, Weijiang Zhang

**Affiliations:** School of Civil and Hydraulic Engineering, Ningxia University, Yinchuan, 750021 Ningxia China

**Keywords:** Rainwater harvesting systems, Soil moisture, Precipitation, Spatial distribution, Apricot tree, Loess Plateau, Ecology, Hydrology

## Abstract

Rainwater is the main water source in arid and semiarid areas of the Loess Plateau, where rainfall is generally insufficient, ineffective and underutilized during the growing season. Thus, improving rainwater utilization efficiency is essential for sustainable agricultural development. A new system composed of rainwater harvesting, an infiltrator bucket with multiple holes and mulching (RHM), was designed to maintain soil moisture at a proper level in rain-fed orchards in arid and semiarid areas of the Loess Region of China. However, there is a lack of clarity on the effectiveness of RHM. In this study, changes in the soil environment and the growth and physiology of apricot trees were monitored via two treatments: (1) Rain-harvesting irrigation system (RHM) treatment and (2) traditional orchard treatment (CK) as a baseline. The results showed that (1) RHM could effectively improve soil water storage at depths of 0–45 cm and at a horizontal distance of 40 cm from the trunk. For the 1.4 mm light rain event, the soil water content increased by 6.3–12%, and for the two moderate rains, the soil water content increased by 12–25%. The change in the soil relative water content predicted by the LSTM model is consistent with the overall trend of the measured value and gradually decreases, and the prediction accuracy is high, with an error of 0.65. (2) The average soil temperatures at 5 cm, 20 cm and 40 cm under RHM were 17.0% (2.4 °C), 13.6% (1.9 °C) and 7.5% (1 °C) greater than those under CK, respectively. (3) Compared with the control treatment, RHM improved the growth and WUEL of apricot trees. The results highlighted the efficiency of the RHM system in enhancing the soil environment and regulating the growth and physiology of apricot trees, which has greater popularization value in arid and semiarid areas.

## Introduction

Soil moisture is a key factor in regulating the soil‒plant-atmosphere continuum and is important in the water cycle in arid and semiarid regions^1,2^. Particularly, in arid and semiarid regions, precipitation is an important source of soil moisture, and the effects of different levels of precipitation on soil moisture vary widely^3,4^. There is a growing gap between the available irrigation water supply and agricultural demand^5^.

Hongmei apricot is a national geographical indicator in China, especially in Ningxia, where the planting area is gradually expanding because of suitable climatic conditions. Traditional rain-fed agriculture has always been the main mode of agricultural production in the region and is mainly restricted by severe water shortages^6^. On the Loess Plateau, the growing season of apricots ranges from March to June, but more than 60% of the annual precipitation falls between July and September^7^. This discrepancy between the rainy season and the apricot growth cycle means that a lack of precipitation probably inhibits apricot production during the early stages, reducing yield at later stages. Water deficiencies at different growth stages diversely affect apricot growth given differences in sensitivity to drought^8,9^. Therefore, the key to increasing agricultural productivity lies in the maximal utilization of precipitation, which requires harvesting light rainfall^10^. It is extremely urgent to implement effective water management measures to fully enhance the efficiency of limited water.

To solve the problems of water scarcity and low water use efficiency, rainfed cultivation techniques have been proposed and gradually improved worldwide^11,12^. The technology of rainwater harvesting and water saving in dryland agriculture is comprehensive, including rainwater harvesting, increasing soil effective capacity, reducing soil evapotranspiration and realizing water saving of crops^13,14^. According to the different methods of implementation and the scale of rainwater harvesting, there are two major types of rainwater harvesting agriculture: Runoff field rainwater harvesting and field micro rainwater harvesting^9^. Runoff field rainwater harvesting involves the transfer of runoff generated in local or small watersheds into a water storage system for use in the dry season^15^, such as water storage cellars, waterlogged ponds, terraces and silt dams^16^. Field microrunoff harvesting is the process of directing collected surface runoff into the soil of the plant root zone by creating a microcatchment area in the field to supplement the water required for crop growth in situ, improve the effective utilization of rainfall, and effectively increase rainfall infiltration recharge^17^. Microrainwater harvesting measures mostly include ridge-furrow planting (RFM)^18^, fish-scale pit planting (FSP)^19^, rainwater collection and infiltration (RWCI)^20^, and storage pit irrigation (WSP)^21^.

For example, ridge-furrow rainwater harvesting can effectively regulate soil water deficit, increase soil water storage in the plow layer and significantly improve water use efficiency in the critical period of crop growth, with obvious yield-increasing advantages^6,9^. Guo et al.^22^ reported that FSP can reduce runoff erosion, significantly increase soil moisture in pits, and improve the vegetation survival rate. The RWCI system has the advantages of saving water, preserving soil moisture and reducing drought stress but performs better than FSP at conserving and utilizing rainwater resources and conserving rhizosphere soil water to meet the water demand of commercial apple orchards^23^. The WSP irrigation method also has the advantages of saving water, maintaining soil moisture, increasing drought resistance, and storing local rainfall runoff^21^. However, these methods are aimed at apples and other crops, such as maize^9^, wheat^24^, and potato^25^, and may not be suitable for apricots. This is because the primary period of water shortage for apricots is from March to May, which differs from the growth season of apples, which occurs from April to October^26^. Furthermore, the interception area of the RWCI system is limited, which causes it to be unsuitable for collecting small amounts of rainfall and less effective during light rainfall. Furrow planting and fish-scale methods are not recommended because they affect only shallow soil moisture.

In response to the development status of the apricot economic forest in the study area and the characteristics of ineffective precipitation being unable to reach the crop roots of the tree, a large loss of evapotranspiration and a low effective utilization rate of precipitation in the area, we proposed a rainfall harvesting irrigation method (RHM). The RHM can realize the accumulation and redistribution of limited rainfall in a specific space of farmland, implement root-zone water replenishment for crops, and improve rainfall utilization efficiency to ensure the production of apricot. Moreover, it has low cost and simple technology and is more easily understood and accepted by local farmers; thus, it has broad application prospects in fruit forest irrigation in arid and semiarid areas of China. However, the soil water and heat changes caused by the application of this technology and its impact on trees have not been studied in depth.

Therefore, our major objectives of this study were to (1) analyze the water transport law in the soil profile of this technology under different types of rainfall conditions and to investigate the effect of this technology on increasing temperature and moisture and (2) explore an efficient way to enhance rainwater management and improve the efficiency of rainfall use in arid/semiarid areas. This information has guiding significance for the promotion and application of this technology in arid and semiarid areas.

## Materials and methods

### Experimental area

A 2-year field experiment was carried out from 2021 to 2022 at the Hongmei apricot orchard of Ningxia Hongmei Apricot Technology Development Co., Ltd., Pengbu Town, Yuanzhou District, Guyuan City, Ningxia Hui Autonomous Region, China (E106° 07′, N36° 08′). The experimental site is in a semiarid region in the west-central part of the Loess Plateau in northwestern China. It has an average altitude of approximately 1750 m and belongs to the inland warm temperate semiarid climate. The weather is cold and dry in winter and spring, and droughts are common in summer. The average annual evaporation ranges from 800 to 1200 mm, while the average annual precipitation ranges from 300 to 400 mm. The rainfall is uneven, with most occurring from July to September (60–70% of the total annual precipitation). The average annual temperature is 6.75 °C, the average sunshine hours are 2250–2700 h, and the frost-free period is approximately 120 days. The soil in the experimental area is sandy loam, and the soil physical properties are shown in Table 1.

**Table 1:** Physical parameters of the experimental soil.

Soil layer (cm)	Clay (%)	Silty sand (%)	Sand (%)	Soil bulk weight (g/cm^3^)	Porosity (%)
0–10	10.48	35.62	53.90	1.57	40.81
10–20	7.50	30.16	62.34	1.66	37.36
20–30	8.51	31.36	60.13	1.65	37.62
30–40	8.99	34.57	56.44	1.68	36.69
40–50	7.76	28.42	63.82	1.67	36.96

### Experimental design and treatments

**Figure 1:**
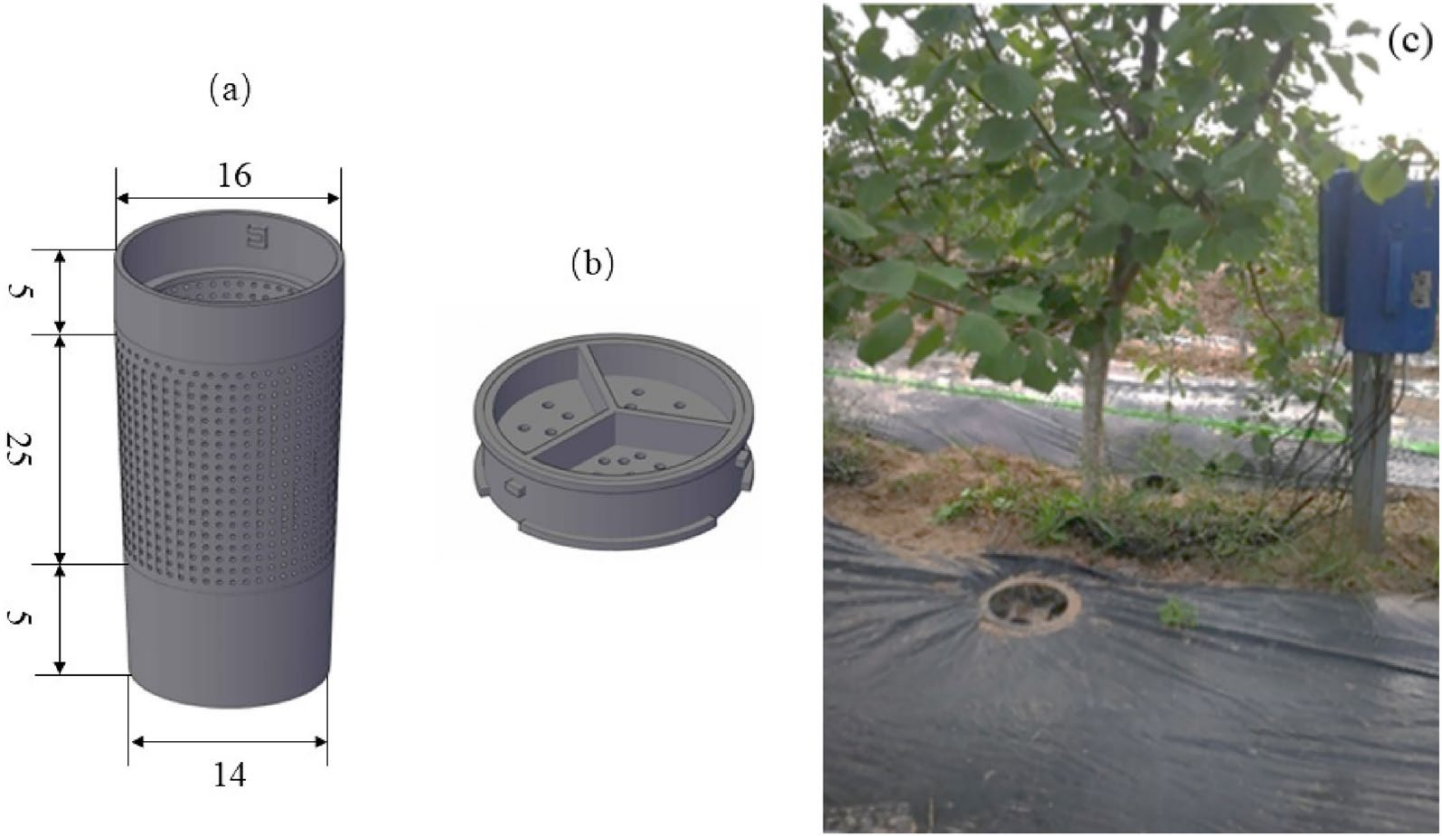
(**a**) Infiltrator bucket diagram (cm), (**b**) infiltrator lid diagram, and (**c**) rainfall-harvested system (RHM).

The experimental orchard area was 40 m × 50 m, and the row spacing was 2 m × 2.5 m. The RHM system was based on the trunk of apricot trees as the center. Along the line of fruit trees, a black polyethylene film 1 m wide and 0.13 mm thick was used as a soil surface covering both sides of the trunk, with a coverage rate of 80%. Infiltration irrigation devices were also buried 50 cm from either side of the trunk. The top of the buried location is lower than the surrounding field surface, which is conducive to water collection. The irrigator was developed independently (Fig. 1a,b). There were two treatments—rainfall catchment infiltration irrigation (RHM) and the control without irrigation but without mulching (CK)—with three replications for each treatment, and the experimental site layout is shown in Fig. 1c. The experiment was initiated in September 2019 to collect rainfall for irrigation of 3-year-old apricot trees, and the experimental data from 2021 to 2022 are presented in this paper.

### Methods and measurements

#### Precipitation

**Figure 2:**
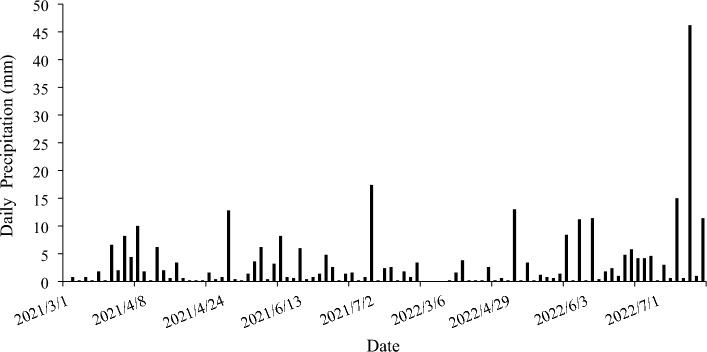
Daily precipitation in Pengbu town from March to mid-July 2021 and 2022.

Daily precipitation was collected by rain barrels to measure the effect of precipitation on soil moisture (Fig. 2).

#### Soil moisture

Soil volumetric water content was measured by an EC-5 (DECAGON, METER, USA) soil moisture sensor, which was set to be recorded every 5 min, and an EM50 recorder compatible with the EC-5 was used for data collection.

Soil moisture sensor burial: We took the top edge of the irrigator as the observation origin, and extended horizontally along the observation origin in the direction from the irrigator and monitor 0–40 cm. Then, we vertically extended downward along the observation origin and monitor 0–45 cm. The sensors were arranged in a 10 × 10 cm grid with 5 layers, and each layer had 4 monitoring points for a total of 20 monitoring points. The first monitoring point on the first layer of the monitoring points was L10D5 (L is the horizontal direction, D is the depth), followed by L20D5, L30D5, and L40D5 from the irrigator direction. In the CK treatment, only the soil water content was monitored at L30D15, L30D25, L30D35, L50D15 and L50D25, and the soil water content was continuously monitored at each monitoring point.

The soil water storage ($W$, mm) was calculated as follows:

1 $$W = \theta_{v} \times h,$$

2 $$\Delta W = W_{i} - W_{i - 1} ,$$

where *θv *(m^3^m^–3^) is the average soil volumetric moisture content; h is the soil layer thickness, 450 mm; and $\Delta W$ is the soil water storage in the period, which is the end of the period $W$ minus the beginning of the period $W$.

#### Calculation of relative water content

The relative soil water content has been used as an agricultural drought index to measure the degree of drought in orchards. It is generally accepted that drought is most severe when the soil relative water content is less than 30%; 30–40% is severe drought; 40–50% is moderate drought; 50–60% is mild drought; and > 60% is no drought^27^.

The soil relative water content (${RWC}_{V}$, %) was calculated as follows^28^:

3 $$RWC_{V} = WC_{{_{V} }} /FC_{V} \times 100,$$

where ${WC}_{V}$(%) is the soil volumetric water content and ${FC}_{V}$ (%) is the field capacity.

#### Soil temperature

During the growing season, the soil temperature dynamics at depths of 5, 20, and 40 cm were continuously monitored using data loggers (JL-04, China) from March to May, and data were collected every 15 min.

#### Physiological and ecological indicators of the apricot trees

##### Leaf photosynthesis

Leaf physiological parameters were measured on sunny days in the stone hardening stage and expanding stage of 2021 with a Li-6400 portable photosynthesis system (Li-COR, Nebraska, USA). In each experimental tree, four sunlit healthy leaves were randomly selected from different directions and tagged to measure the leaf physiological parameters at 14:00. Each leaf was measured three times, after which the leaf water use efficiency (WUEL) was calculated as the net photosynthetic rate (Pn) divided by the transpiration rate (Tr).

##### Relative chlorophyll content and tree growth

Four leaves and branches in the four directions of the three trees were selected and tagged for each treatment. The leaf area (LI-3000c, LI − COR, Nebraska, USA), SPAD (SPAD 502Plus, Konica Minolta, Japan), and branch length (tape) were measured in May (stone hardening stage), June (expanding stage), and July (mature stage), respectively. Because the height, crown width and round diameter of the trees grew slowly during the growth period, only 40 trees (in the mature period) were randomly selected in July and measured with Vernier calipers, tape measures, and custom-made rulers (marked on a 6 m long steel rod).

### Data analysis

Data analysis and calculations were performed using IBM SPSS Statistics25, and figures were created using Excel 2019 and Origin 2018. The predictions were simulated using LSTM, and the root mean square error (RMSE) was used to assess the agreement between the measured and predicted values. Statistically significant differences between means were determined using the least significant difference (LSD) 0.05 test, and medians were analyzed for trends in data concentration.

### Plant research statement

Experimental research and fieldwork on plants (cultivation), including collection of plant material, were conducted in compliance with relevant institutional, national and international guidelines and regulations.

## Results

### Characteristics of single rainfall events in different periods

The apricot plants were susceptible to drought stress due to low rainfall in March–May. We mostly analyzed four small rainfall events in March–May 2021, which were classified as two small and two medium rainfall events according to the standard of ‘Precipitation Rating (GB-T28592-2012)’ (Table 2), and all occurred during the period of insufficient precipitation, one week before or after the rainfall without precipitation.

**Table 2:** Rainfall distribution characteristics of the four rainfall events at the test site.

Rainfall events	I	II	III	IV
Date of rainfall	3.9–3.10	3.18–3.19	5.1	5.9
Rainfall (mm)	1.2	8.6	8	1.4
Average rainfall intensity (mm/min)	0.04	0.04	0.07	0.2
Maximum rainfall intensity within 30 min (mm/min)	0.6	2.2	3	1.4
Rainfall level	Light rain	Medium rain	Medium rain	Light rain

Rainfall events I and IV were light rain events. Rainfall I was mostly characterized by a long duration and low rainfall intensity, and rainfall IV was characterized by a short duration and medium intensity. Rainfall events II and III were moderate rainfall events, where rainfall event II was long-duration rainfall with medium intensity, and rainfall event III was short-duration rainfall with high intensity; other parameters are shown in Table 2.

### Soil moisture dynamics under different rainfall conditions in the RHM treatment

**Figure 3:**
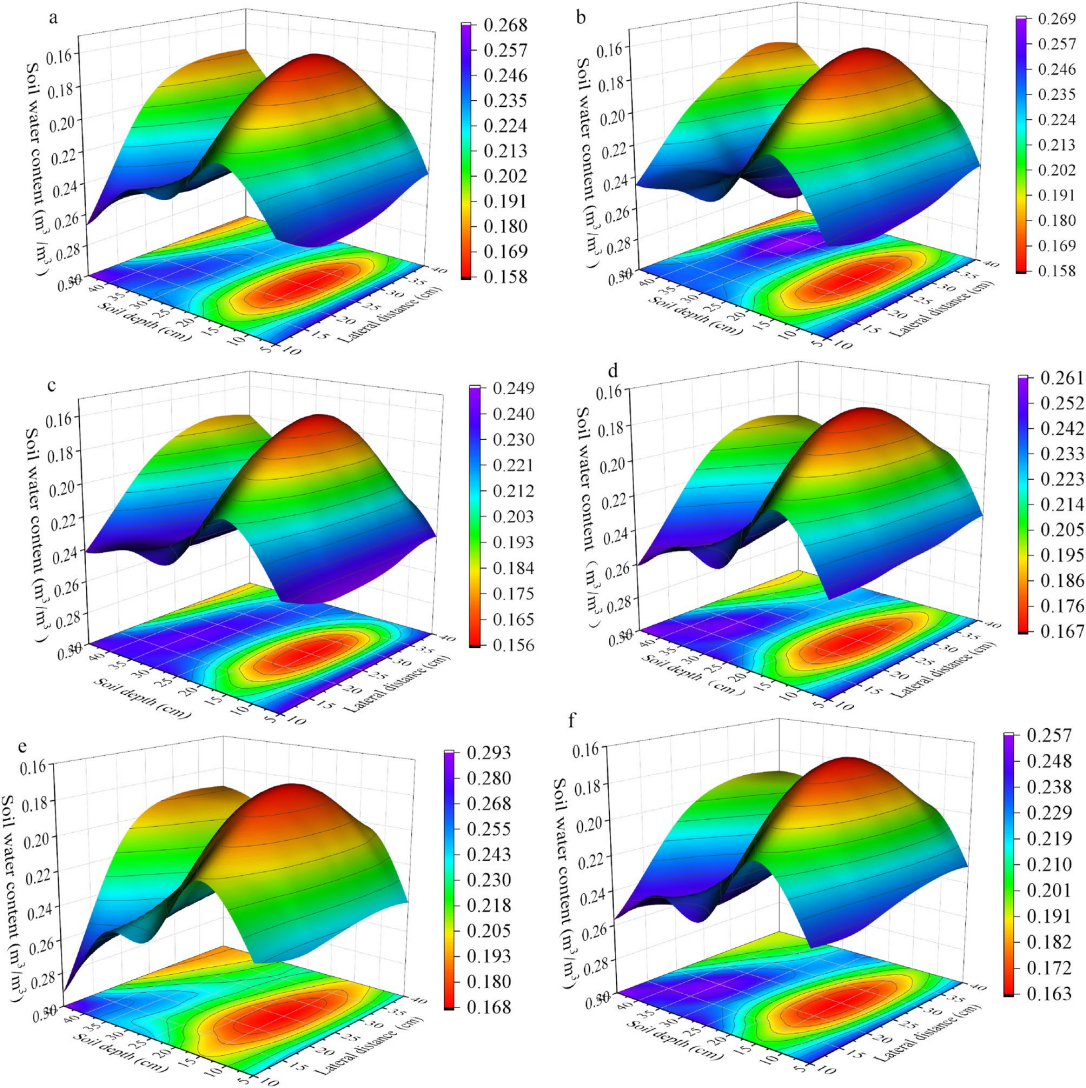
Soil moisture dynamics of RHM during light rainfall events. (**a**) Before event I rainfall, (**b**) during event I rainfall, (**c**) after event I rainfall, (**d**) before event IV rainfall, (**e**) during event IV rainfall, and (**f**) after event IV rainfall.

**Figure 4:**
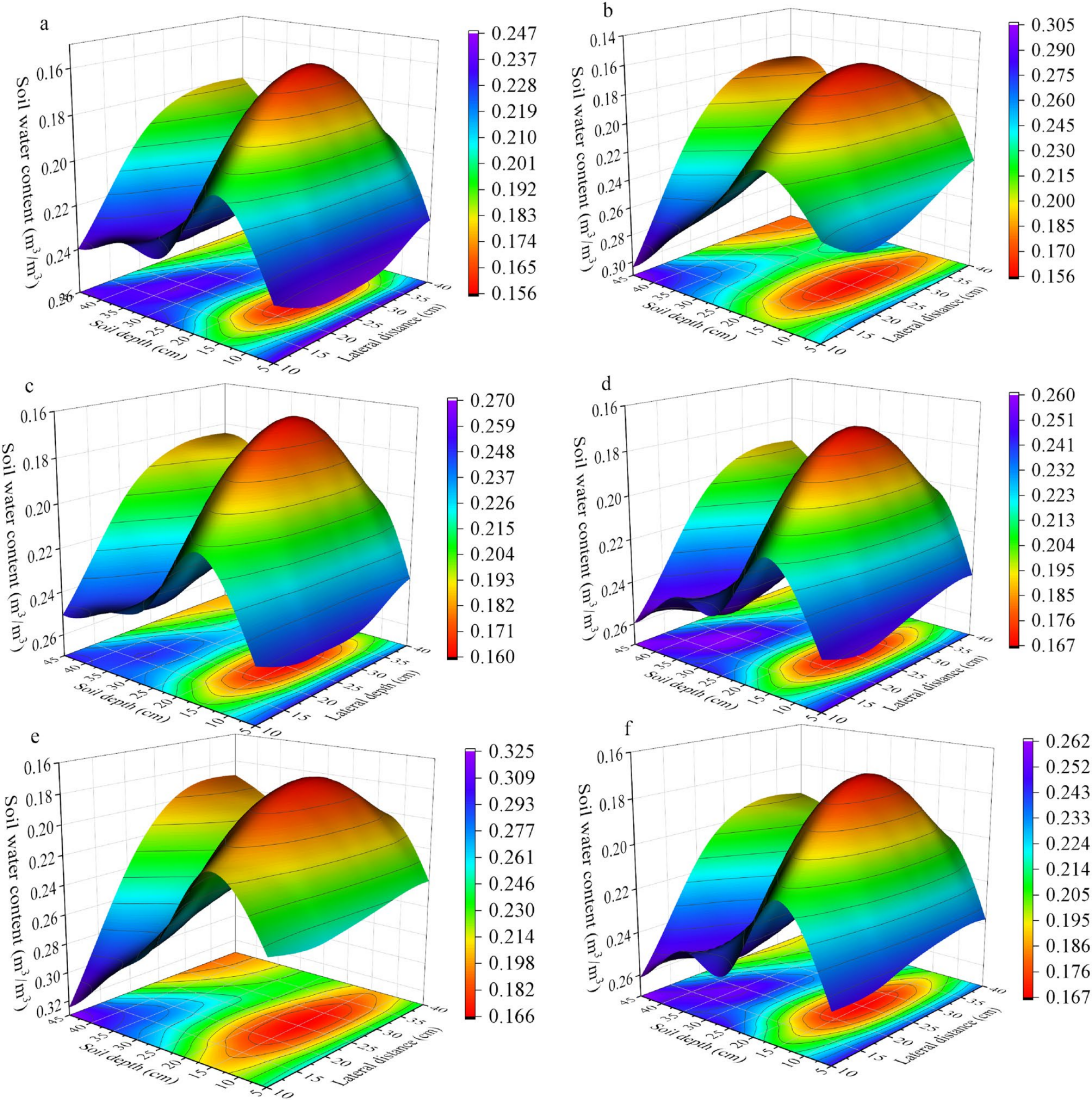
Soil moisture dynamics of the RHM during moderate rainfall events. (**a**) Before event II rainfall, (**b**) during event II rainfall, (**c**) after event II rainfall, (**d**) before event III rainfall, (**e**) during event III rainfall and (**f**) after event III rainfall.

In the light rainfall event, SMC (soil moisture content, m^3^m^–3^) was analyzed one day before, during and three days after the rainfall. While in the medium rainfall event, SMC was analyzed one day before, during and seven days after the rainfall. The average vertical and horizontal distributions of the SMC during this study period for the four rainfall events exhibited significant differences, with consistent spatial characteristics for the different rainfall events under the same treatment, as shown in Figs. 3 and 4. There were two soil layers (0–5 cm and 20–45 cm) that had greater SMC, 40 cm from the trunk, in the RHM treatment (Figs. 3, 4). Before rainfall, the average soil water content of event I was 0.202–0.268, and that of event IV was 0.205–0.261, with no significant difference. Notably, during the rainfall period, the soil moisture content of event I in this range was between 0.202 and 0.269 (Fig. 3b), which changed only slightly. Compared with that of event I, the soil moisture of event IV began to gradually spread outward with the centre of catchment infiltration, and the soil moisture content at each depth and horizontal gradually increased. In particular, soil moisture in the 20–45 cm depth and 10 − 40 cm horizontal distance was noticeably replenished, and the SMC increased to 0.218–0.293 (Fig. 3e), which was an increase of 6.3–12.3%.

Rainfall events II and III showed a similar vertical SMC distribution as that of the two light rainfall events for the 0 − 45 cm soil layer 40 cm from the trunk. In event III, compared with that in the prerainfall period, the soil moisture at horizontal 0–40 cm was relatively high (increased 12.8–25%) in the 0 − 45 cm soil layer. In event II, the increase was 12.0–23.5%, and the increase in event III during rainfall was greater than that in event II. The soil moisture gradually decreased seven days after the end of rainfall and was 0.49 − 0.77% (rainfall III) and 5.88 − 9.31% (rainfall II) greater than that before rainfall.

### Soil moisture dynamics before and after a single rainfall event in the CK treatment and comparison with the RHM

**Figure 5:**
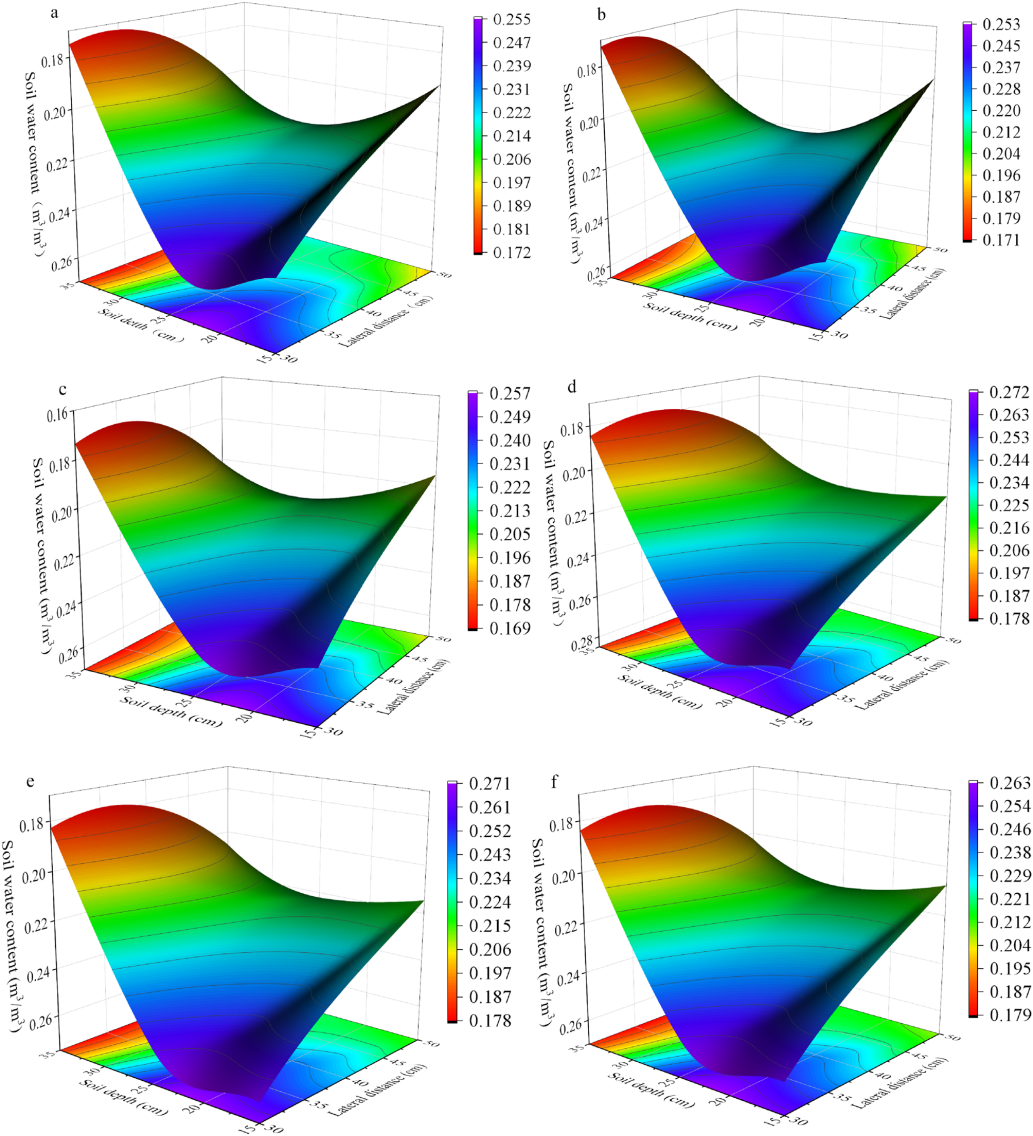
Soil moisture dynamics of CK during moderate rainfall events. (**a**) Before event II rainfall, (**b**) during event II rainfall, (**c**) after event II rainfall, (**d**) before event III rainfall, (**e**) during event III rainfall and (**f**) after event III rainfall.

The analysis and comparison of soil moisture dynamics during two moderate rainfall events in the RHM and CK treatments are presented in this study. Figure 5 shows that the distribution characteristics of soil moisture in the CK treatment were similar to those in the RHM treatment during the two rainfall events. Soil moisture was mainly distributed at depths of 0–30 cm and horizontal distances of 0–40 cm, which roughly corresponds to a quarter of an ellipse. The figure shows a clear difference in the distribution of soil moisture content (SMC) between the two methods, as seen from the soil water content before and after rainfall. None of the CK treatments exhibited a significant increase in SMC compared to that in the prerainfall period.

### Changes in the relative soil moisture content in the wet layer of the plan after rainfall

The relative SMC was calculated at horizontal distances of 0–30 cm and at depths of 25, 35 and 45 cm at the end of light rainfall event IV (1.4 mm) (Table 3). The results showed an increase in the relative SMC within the range of lateral distance of 0–30 cm and depth of 25–45 cm, except at monitoring point L30D45. As the root system of the studied apricot trees is mainly distributed laterally within depths of 0–40 cm and 25–35 cm, we analyzed the moisture conditions around the root system using the relative SMC of L10D25 (40 cm from the trunk). In this paper, to determine the degree of drought, we used the LSTM memory network to predict the trends in the relative water content of L10D25 over time, which can be supplemented over time to prevent soil drying.

**Table 3:** Comparison of relative soil moisture content in different soil layers before and after rainfall.

Lateral distance (cm)	25			35			45		
	Θb	Θar	RE	Θb	Θar	RE	Θb	Θar	RE
10	144.46	147.49	3.03	140.18	150.22	10.04	155.12	177.79	22.67
20	124.77	126.79	2.02	146.71	150.22	3.52	128.33	131.94	3.61
30	131.33	133.35	2.02	130.13	130.64	0.50	115.97	115.45	− 0.52

Θb is the relative water content before rainfall, Θar is the actual relative water content after rainfall, and RE is the increase in %.

The dataset was first aggregated from 5-min intervals to hourly datasets, resulting in a total of 1327 rows after the rain stopped. To ensure consistency between the network prediction time step and the actual time scale of the soil, the soil relative water content values of consecutive equal time intervals were formed into a sequence with a length of 1327 equal time steps. To facilitate the analysis of the prediction results, the first 1138 samples of the soil relative water content sequence were used as the training set for model training and adjustment of the model parameters, and the remaining 189 samples were used as the model testing set for error estimation of the prediction results and model evaluation.

**Figure 6:**
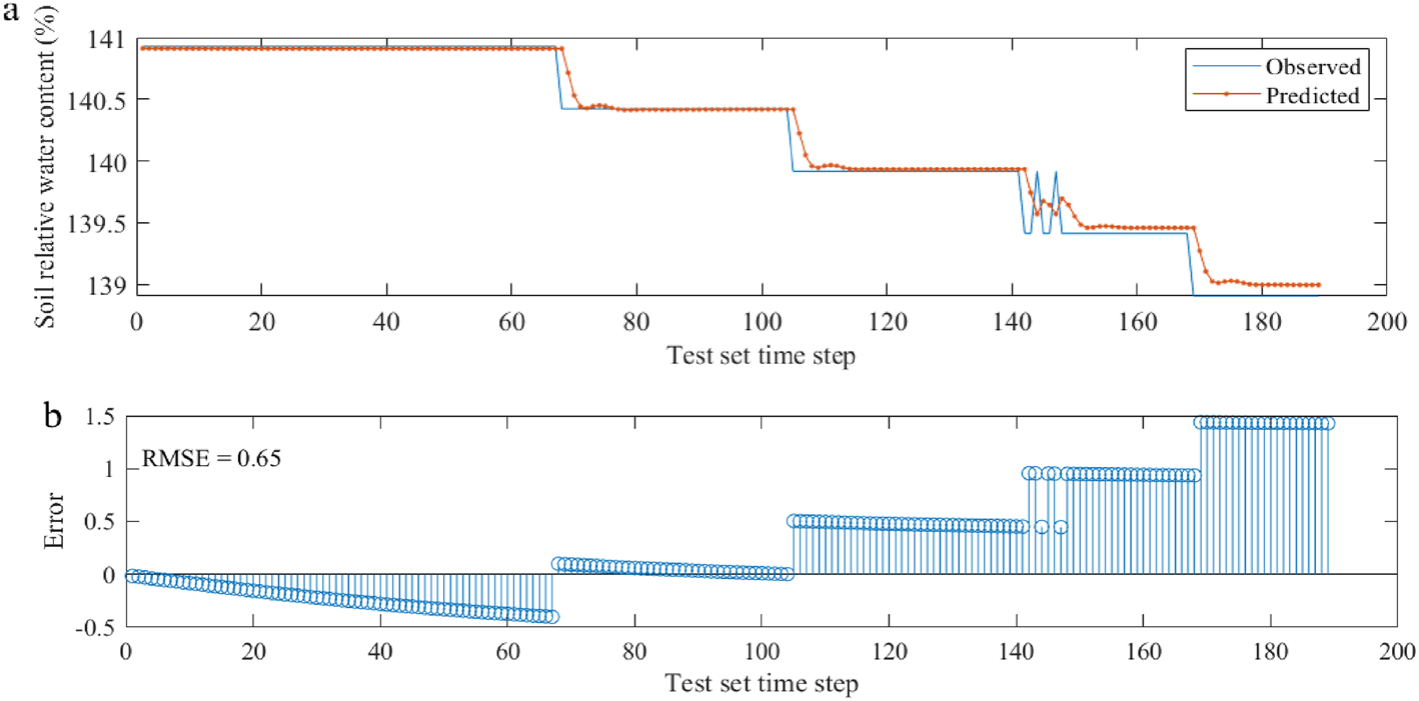
Prediction of relative soil moisture content and prediction error.

The soil relative water content prediction curve of L10D25 is shown in Fig. 6a. The simulation results during the training period of the model were better, with an RMSE of 0.65 and an error ranging between -0.5 and 1.5% (Fig. 6b), and the soil relative water content decreased from 147.5–139%.

### Effect of the rainfall catchment infiltration system on the soil temperature

#### Soil temperature changes during the growth period of apricot trees

**Figure 7:**
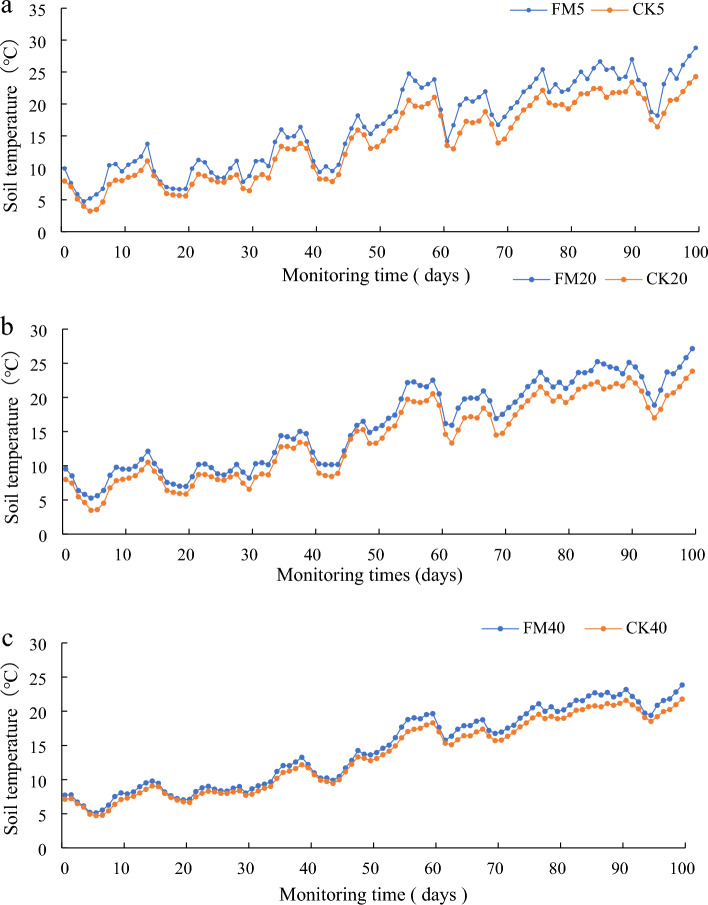
Dynamic changes in soil temperature in apricots from March to June. (**a**) 5 cm; (**b**) 20 cm; (**c**) 40 cm.

Figure 7 and Table 4 show the soil temperature dynamics at depths of 5, 20 and 40 cm during the growth period of the apricot trees. The warming effect of soil temperature was similar. Furthermore, the soil temperatures in the different treatments were influenced by weather conditions. The results showed that the RHM treatment increased the average soil temperature during the growing period of the apricot trees, with the most significant increase observed at a depth of 5 cm. Although the variation trend of soil temperature was similar, soils at different depths showed higher temperatures under the RHM treatment than under the CK treatment. The warming effect of mulching also varied during growth periods. During the early stage of apricot tree growth, the soil temperatures at three locations under the RHM treatment were 20.2% (1.8 °C), 14.6% (1.3 °C) and 7% (0.6 °C) greater than those under the CK treatment at depths of 5, 20 and 40 cm, respectively. In the late stages of apricot tree growth, the difference between the two treatments gradually increased with increasing temperature. Specifically, the RHM was 16.2% (3.1 °C), 12.1% (2.3 °C) and 7.2% (1.3 °C) greater than that in the CK treatment, respectively. Throughout the growth period, the soil temperature in the RHM treatment was consistently greater than that in the CK treatment, with increases of 17.0% (2.4 °C), 13.6% (1.9 °C) and 7.5% (1 °C), respectively.

**Table 4:** Average soil temperature in the 5, 20, and 40 cm soil layers under different treatments during apricot tree growth periods.

Soil depth (cm)	Treatment	TE (°C)	TL (°C)	TG (°C)
5	RHM	10.7 ± 3.3b	22.2 ± 3.2b	16.5 ± 6.6b
	CK	8.9 ± 3.0b	19.1 ± 2.8b	14.1 ± 5.9b
20	RHM	10.2 ± 2.7b	21.3 ± 2.8b	15.9 ± 6.2b
	CK	8.9 ± 2.8b	19.0 ± 2.7b	14.0 ± 5.8b
40	RHM	9.2 ± 2.2a	19.4 ± 2.5a	14.4 ± 5.6a
	CK	8.6 ± 2.1a	18.1 ± 2.3a	13.4 ± 5.3a

Means followed by different letters are significantly (p < 0.05) different between RHM and CK.

*TE* early temperature, *TL* late temperature and *TG* growth period temperature.

#### Characterization of daily soil temperature changes

**Figure 8:**
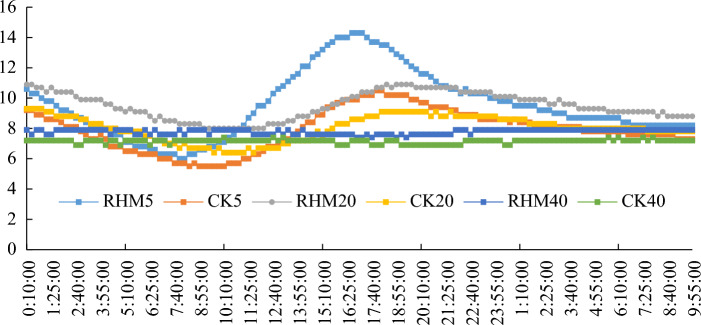
Diurnal variation of soil temperature.

The daily variation in soil ground temperature from March 15 to March 16 was selected for analysis. The different treatments had varying effects on the diurnal variation in soil temperature at the different time points (Fig. 8). The time lag effect of the soil temperature response to air temperature increased with soil depth, and its fluctuation was gradually reduced by solar radiation. The magnitude of the change decreased, and the magnitude of the daily change in soil temperature at 40 cm was very small. The temperature of the RHM 20 treatment was the highest from 0:00 a.m. to 4:00 a.m. Except at a depth of 40 cm, the soil temperature of the other soil layers gradually increased at approximately 9:00 am and peaked at approximately 16:40, and the soil temperature of the shallow layer was higher than that of the deep layer. Mulching altered the surface wavelength radiation coefficient and regulated the soil heat absorption conditions so that the soil temperatures in the mulched treatment were significantly higher than those in the nonmulched treatment. The RHM5 treatment reached a maximum of 17.2 °C, 24.4% higher than that of the CK5 treatment, but the soil temperature at a depth of 40 cm changed only slightly, and that of the RHM40 treatment was 10.5% higher than that of the CK40 treatment. During the period of continuous warming, the soil temperature basically decreased as follows: RHM5 > CK5 > RHM 20 > CK20 > RHM 40 > CK 40. After 16:40, the temperature and solar radiation decreased sharply, which caused the shallow soil temperature to decrease significantly, while the deep soil temperature changed slowly due to the time lag effect.

### Leaf photosynthesis

Table 5 shows the soil water storage and photosynthetic parameters of the apricot leaves under the different treatments during the stone hardening stage and expansion stage. The water storage of RHM was much greater than that of CK. Under the same rainfall conditions, Pn, Tr, Gs and RH (relative humidity) gradually decreased with decreasing soil water storage. The maximum values of these four photosynthetic parameters usually occurred under RHM, and the minimum values occurred under CK. Ci exhibited the opposite trend, and the Ci of the CK treatment was the greatest, indicating that the photosynthesis rate of the CK treatment was low. The Pn, Tr and WUEL were 5.94 μmol m^–2^ s^–1^, 3.02 mmol m^–2^ s^–1^ and 2.09 μmol mmol^–1^ at the stone hardening stage, which were 80.5% (3.29 μmol m^–2^ s^–1^), 45.9% (2.07 mmol m^–2^ s^–1^) and 31.4% (1.59 μmol mmol^–1^) higher than those in the CK treatment, respectively. However, the difference gradually decreased in the expanding stage, and the three indexes under RHM were 17.1%, 14.7% and 2.8% higher than those under CK, respectively.

**Table 5:** Soil water content, net photosynthetic rate (Pn), transpiration rate (Tr), stomatal conductance (Gs), internal CO_2_ concentration (Ci), relative humidity (RH) and water use efficiency (WUEL) of apricot tree leaves under different treatments.

Growth period	Treatments	ΔW	Pn	Tr	Gs	Ci	RH	WUEL
Stone hardening stage	RHM	32.4	5.94 ± 0.11	3.02 ± 0.12	0.09 ± 0.01	258.28 ± 2.58	22.5 ± 1.09	2.09 ± 0.03
	CK	− 4	3.29 ± 0.08	2.07 ± 0.13	0.06 ± 0.02	292.87 ± 1.69	21.03 ± 1.23	1.59 ± 0.09
Expanding stage	RHM	43.5	10.9 ± 0.12	3.27 ± 0.08	0.10 ± 0.03	201.75 ± 5.21	36.43 ± 1.55	3.35 ± 0.11
	CK	− 9	9.31 ± 0.07	2.85 ± 0.51	0.10 ± 0.02	204.03 ± 4.23	39.55 ± 1.36	3.26 ± 0.05

### Effects of RHM on apricot tree growth

**Figure 9:**
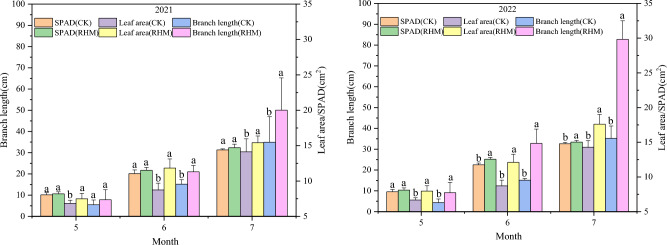
Comparison of the SPAD values, leaf areas and branch lengths of apricot trees subjected to different treatments from May–July 2021–2022. Means followed by different letters are significantly (p < 0.05) different between RHM and CK.

RHM greatly increased the SPAD, leaf area, and branch length of the apricots from May–July across the two studied years (Fig. 9). Compared with the CK treatment, the RHM treatment had 0–4.7%, 51.1–58.2% and 45.5–104.9% greater SPAD, leaf area and branch length values, respectively, in May. Additionally, the SPAD, leaf area, and branch length under RHM were 7.7–11.8%, 48.3–59.3% and 38.5–143.8%, respectively, in June and 2.5–3.3%, 13.8–24.6% and 66.1–139.2%, respectively, in July compared with those under CK. Thus, RHM treatment may have enhanced apricot growth.

**Figure 10:**
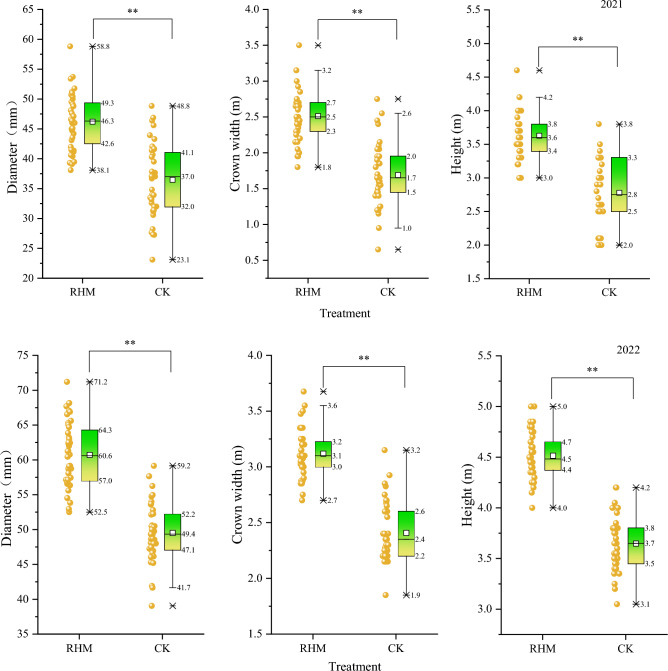
Comparison of ground diameter, crown width, and height among treatments of apricot trees, 2021–2022. Different lower-case letters for values in the same row indicate significant treatment differences at P < 0.05 using the LSD test.

Figure 10 shows the differences in tree height, crown width and diameter of apricot trees under different treatments. The mean values of these were 21.07%, 32.77% and 23.43% higher than the control treatment in 2021 and 22.3%, 23.6% and 29.5% higher in 2022, respectively, indicating that RHM promoted the growth of apricot trees. The narrower box of basal diameter, crown spread and tree height of RHM and crown spread of CK indicated that the data distribution was more concentrated and the degree of fluctuation was small, while the distributions of tree height and ground diameter of CK treatments were more dispersed. The maximum values of basal diameter, crown width and tree height all occurred in the RHM treatment.

## Discussion

### Soil moisture

Due to the uneven distribution of precipitation on the Loess Plateau, the supply of water resources is sometimes insufficient to maintain the normal growth of crops. In addition, summer crops often suffer from drought during the growth period due to soil water deficit^29^. In this region, the rainfall is low, and the evaporation is strong, resulting in large fluctuations in the soil water content. Compared with traditional tillage, the ridging and plastic film mulching techniques can collect light rainfall and increase the soil water content in the plant root zone, and they are more effective at improving crop yield, water use efficiency and economic benefits^30,31^. Therefore, three major factors could influence the spatial distribution of soil moisture, rainfall, film mulching and infiltration irrigation devices in this study.

In this study, we compared four types of rainfall and revealed that the RHM treatment significantly affected short-duration light rain (1.4 mm) and moderate-duration rain (8.6 and 8 mm). Compared with that before rainfall, the soil water content at 0 − 45 cm depth in the root zone of red plum apricots increased. However, the soil water content did not increase significantly during event I (Fig. 3b). This may be due to the long duration of rainfall and low intensity of rainfall event I, as well as canopy interception and evaporation, which prevented the formation of runoff or small amounts of runoff and hindered the collection of rainwater. This finding is similar to that of Li et al.^32^, who reported that more than 1 mm of rainfall could generate effective runoff from plastic film mulch, thereby increasing soil moisture. On the other hand, mulching can significantly improve the effectiveness of soil moisture by reducing soil evaporation loss and increasing soil water storage^6^. Our results revealed that the soil water content (SMC) in the 0 − 45 cm soil profile was significantly greater under the mulching treatment than under the control treatment (Fig. 5). The four rainfall events did not cause an increase in soil moisture, which could be attributed to the absence of plastic film mulching, which led to a large evaporation loss of soil moisture due to the direct impact of solar radiation. The RHM systems can significantly increase the extent of wetted areas in the spatial profile. The recharge effect of the RHM system was most prominent at 0–5 cm and 20 − 45 cm soil depths and 0 − 40 cm from the trunk. The possible cause of this result is that rainwater flows directly into the infiltration bucket through a multiholed bucket wall, in which the infiltration bucket is placed relatively deep, resulting in insufficient soil moisture replenishment in the upper soil layer (5 − 20 cm). Furthermore, the RHM treatment supplemented water to the lower SMC area, which was driven by root absorption (Figs. 3c and f, 4c and f).

Drought is a prolonged dry and stable climatic phenomenon that is a manifestation of water-heat imbalance caused by a lack of sufficient precipitation. SMC estimation is active in weather forecasting, climate research, soil and water conservation and the implementation of sustainable agricultural management practices^33^. For example, irrigation scheduling should be carried out, and the appropriate tillage time should be determined. The LSTM model is widely used in soil moisture prediction^34^, and the soil relative water content is widely used as an indicator of the degree of drought in agricultural planting^35,36^. In this study, the soil relative water content in the main distribution area (25 cm soil layer) of apricot roots under RHM treatment of light rain event IV (rainfall 1.4 mm) was calculated and predicted. We found that the relative soil moisture content was much greater than 60%, which indicated that the soil in the RHM treatment with 1.4 mm of rainfall was well hydrated and that there was no drought (Fig. 6). These results demonstrated that the RHM system is useful for efficiently utilizing rainwater resources in rainfed orchards in arid and semiarid regions of the Loess Plateau.

### Soil temperature

Temperature is vital in the growth and development of crops^37^. Plastic film mulching can increase the temperature of the soil surface by reducing the exchange of latent and sensible heat between the soil and surrounding air^38^. The warming effect of plastic film mulching is mainly related to solar radiation heat. Mulching not only affects the water and heat exchange between the soil and atmosphere but also reduces the heat loss caused by soil moisture evaporation^6^. However, the warming effect varies depending on the mulch material, crop type, planting method, growing season and developmental stage of the crop^39^. In this study, the RHM treatment significantly increased the daily average soil temperature of the 0–40 cm layer compared to that of the control for the same soil layer. However, the temperature increase was not significant during the early apricot growth stage. The low seasonal temperature during the early apricot growth stage may be the main reason for the fluctuations in shallow soil temperature changes, while the deep soil temperature remains relatively stable. Table 4 shows that the temperature difference decreases as the soil layer depth increases. However, with increasing air temperature, the trend of surface soil warming gradually increased. This was because the RHM treatment received more solar energy at the soil surface, resulting in the highest topsoil temperature. Similar results were reported by He et al.^40^.

In addition, the diurnal variation in soil temperature is also greatly affected by air temperature (Fig. 8). In terms of the diurnal variation in soil temperature, the soil temperature at 0–20 cm in the two treatments was consistent with the temperature change, but the change at 40 cm was relatively slight. From the temperature of different soil layers in the same treatment, the soil temperature gradually decreased with increasing soil depth. In the present study, the diurnal soil temperature amplitude was greater in the RHM treatment than in the control treatment. The shallow soil temperature fluctuated strongly with the atmospheric temperature^41^. However, its fluctuation lags behind that of the atmospheric temperature because the deep soil receives less solar radiation, the soil layer is deeper, and the temperature fluctuation is smaller. Duan et al.^6^ reported that compared with the CK treatment, the ridge-furrow mulching system reduced the diurnal variation in soil temperature across two study years, which was contrary to our results. This may be due to differences in the distribution and crest characteristics of solar radiation on the soil surface due to differences in the microtopography of ridges and furrows, and increased rainwater received in the furrows could influence diurnal variations in soil temperature. Due to favorable soil temperature conditions, RHM may be the most effective at regulating the growth and physiological characteristics of apricots.

### The ecophysiological characteristics of apricot growth

The morphological, physiological and biochemical characteristics of plants are usually modified by the SMC, subsequently affecting plant growth and development. Photosynthesis provides organic compounds and energy for plants and is a key factor affecting the growth and productivity of fruit trees. RHM effectively improved the photosynthetic efficiency, which may have been due to the improvement in the soil conditions (higher soil moisture and stable soil temperature), which was consistent with the results obtained for winter wheat^37^. In this study, the Pn was greater in RHM than in CK, suggesting that soil moisture deficit reduces the Pn. This conclusion supports the findings of previous studies^42^. Stomata are the passageways of gas exchange between plant leaves and the external environment, and they are also important channels for the evaporation of moisture. It is generally recognized that improved photosynthesis could be determined by stomatal behavior under plastic mulching conditions^43^. We found that the stomatal density of the apricot plants in the CK treatment was lower than that in the RHM treatment (Table 5), possibly because the nonmulched apricot trees experienced drought stress due to increased soil evaporation and a lack of soil moisture replenishment.

Mulching can modulate the relationship between soil moisture and heat and improve root quality, thereby altering crop developmental processes. The diameter will produce expansion or contraction feedback and then produce a slight change in diameter. Monitoring the trunk index has been proven to quickly reflect the impact of water and heat on trees^44^. Researchers have shown that the plant traits of mulching treatments are greater than those of nonmulching^45^. Liu^46^ found that pear trees grow best and healthiest under rain-harvesting conditions, which is similar to our findings. Our results also revealed that compared with the CK treatment, the RHM treatment significantly increased apricot growth. This was attributed to the RHM improving the water and heat conditions, which may have promoted apricot tree root growth and thus nutrient and soil water uptake. The RHM treatments substantially augmented the crown width, height and diameter of the apricot trees and produced the greatest increase; thus, there is much potential to increase apricot yields. Many studies have demonstrated that fine root growth and distribution in the soil profile are positively correlated with changes in the SMC^47^. More water means faster root growth, with more roots concentrated deeper in the higher SMC, and the tree can then adjust its water uptake strategy based on the soil moisture profile within the rhizosphere. As our results demonstrated that RHM enhances the SMC and crop water productivity in apricot orchards, it is necessary to quantitatively evaluate how these management practices affect root growth and development.

## Conclusion

Insufficient rainfall and low utilization limit the production of apricot in southern Ningxia. During the critical period of apricot growth from March to May, RHM increased soil moisture and temperature and reduced soil evaporation by efficiently collecting and utilizing rainwater, thus promoting the growth of apricot trees. During the experiment, compared with the RHM treatment, the RHM treatment resulted in the collection of invalid rainfall, such as light rain with a short duration and high rainfall intensity greater than 1.4 mm and two types of moderate rain. Soil moisture and temperature increased significantly in the 0–45 cm soil layer. Compared with the CK treatment, the RHM treatment significantly improved the water use efficiency of the apricot leaves, promoted the growth of the apricot trees and had great potential to improve the apricot yield. The installation of a rainfall collection and infiltration irrigation device (RHM) can effectively alleviate the problems of drought and water shortages during the growth period of apricot trees and promote the growth of trees while improving the effective utilization rate of rainfall. This planting method significantly improves soil moisture by improving rainwater harvesting efficiency and transpiration while reducing soil evaporation and can be used as a sustainable water management method to help mitigate the negative effects of drought. Therefore, RHM treatment should be considered a highly water-saving method because it can optimize the collection and utilization of rainwater, thereby alleviating the shortage of water resources in Northwest China, and it has greater value in arid and semiarid regions.

## Data Availability

The data presented in this study are available upon request from the corresponding author.
